# RETRACTION: Efficiency of Platelet‐Rich Plasma in the Management of Burn Wounds: A Meta‐Analysis

**DOI:** 10.1111/iwj.70259

**Published:** 2025-02-25

## Abstract

**RETRACTION**: ImamM. S. , AlotaibiA. A. S. , AlotaibiN. O. M. , AlosaimiN. S. , AlotaibiS. G. M. , and AbdelrahimM. E. A. , “Efficiency of Platelet‐Rich Plasma in the Management of Burn Wounds: A Meta‐Analysis,” International Wound Journal 21, no. 2 (2024): e14419, 10.1111/iwj.14419.PMC1082507037776166

The above article, published online on 30 September 2023, in Wiley Online Library (http://onlinelibrary.wiley.com/), has been retracted by agreement between the journal Editor in Chief, Professor Keith Harding; and John Wiley & Sons Ltd. Following an investigation by the publisher, all parties have concluded that this article was accepted solely on the basis of a compromised peer review process. The editors have therefore decided to retract the article. The authors did not respond to our notice regarding the retraction.

**RETRACTION**: M. S. Imam , A. A. S. Alotaibi , N. O. M. Alotaibi , N. S. Alosaimi , S. G. M. Alotaibi , and M. E. A. Abdelrahim , “Efficiency of Platelet‐Rich Plasma in the Management of Burn Wounds: A Meta‐Analysis,” International Wound Journal 21, no. 2 (2024): e14419, 10.1111/iwj.14419.PMC1082507037776166

The above article, published online on 30 September 2023, in Wiley Online Library (http://onlinelibrary.wiley.com/), has been retracted by agreement between the journal Editor in Chief, Professor Keith Harding; and John Wiley & Sons Ltd. Following an investigation by the publisher, all parties have concluded that this article was accepted solely on the basis of a compromised peer review process. The editors have therefore decided to retract the article. The authors did not respond to our notice regarding the retraction.

